# FORTIS: a live-cell assay to monitor AMPA receptors using pH-sensitive fluorescence tags

**DOI:** 10.1038/s41398-021-01457-w

**Published:** 2021-05-27

**Authors:** María Calleja-Felipe, Magdalena Natalia Wojtas, Marta Diaz-González, Dalila Ciceri, Raúl Escribano, Alberto Ouro, Miguel Morales, Shira Knafo

**Affiliations:** 1 Department of Physiology and Cell Biology, Faculty of Health Sciences, The National Institute for Biotechnology in the Negev, and The Zlotowski Center for Neuroscience, Ben-Gurion University of the Negev, Beer-Sheva, Israel; 2 Instituto Biofisika (UPV/EHU, CSIC), University of the Basque Country, Leioa, E-48940 Spain; 3 Fundación Biofísica Bizkaia/Biofisika Bizkaia Fundazioa (FBB), Barrio Sarriena s/n, Leioa, E-48940 Spain; 4 Ikerbasque, Basque Foundation for Science, Bilbao, 48013 Spain

**Keywords:** Clinical pharmacology, Hippocampus

## Abstract

The real-time live fluorescent monitoring of surface AMPA receptors (AMPARs) could open new opportunities for drug discovery and phenotypic screening concerning neuropsychiatric disorders. We have developed FORTIS, a tool based on pH sensitivity capable of detecting subtle changes in surface AMPARs at a neuronal population level. The expression of SEP-GluA1 or pHuji-GluA1 recombinant AMPAR subunits in mammalian neurons cultured in 96-well plates enables surface AMPARs to be monitored with a microplate reader. Thus, FORTIS can register rapid changes in surface AMPARs induced by drugs or genetic modifications without having to rely on conventional electrophysiology or imaging. By combining FORTIS with pharmacological manipulations, basal surface AMPARs, and plasticity-like changes can be monitored. We expect that employing FORTIS to screen for changes in surface AMPARs will accelerate both neuroscience research and drug discovery.

## Introduction

In the central nervous system (CNS), excitatory glutamatergic synapses control neurotransmission mediated by ion flow through α-amino-3-hydroxy-5-methyl-4-isoxazolepropionic acid receptors (AMPARs). Regulating the exo- and endocytosis of AMPAR is a critical aspect of synaptic plasticity, influencing long-term potentiation (LTP) and long-term depression (LTD) at excitatory synapses. Consequently, it is now recognized that AMPARs are crucial for many aspects of brain function, including learning and memory^1–5^, and abnormal AMPAR transmission has been implicated in cognitive impairment^4,6–8^. Pharmacological and molecular manipulations can enhance cognitive function in mice^9–11^, demonstrating that improved synaptic function, including some forms of synaptic plasticity, plays a critical role in cognitive enhancement^12–16^. Indeed, there is evidence that boosting AMPAR activity may be an efficient approach to modulate synapses that undergo experience-dependent changes to enhance cognition^16–19^. Given the importance of AMPARs in brain function, there is a clear need to rapidly screen for drugs and conditions that alter AMPAR expression and function in physiologically relevant contexts.

Synaptic plasticity has for long been investigated using primary cultures of neurons^17,18,20,21^. To date, approaches to monitoring synaptic transmission and synaptic plasticity have focused on both electrophysiology and the imaging of individual dendritic spines. Both methods are well established and supply considerable information regarding synaptic function in a variety of conditions. Nevertheless, such approaches are labor-intensive and low-throughput, and they are not suited to the fast evaluation of drugs or rapid phenotyping. In terms of drug screening and discovery, many assays have been developed based on human neuroblastoma cell lines^22–26^ representing an unlimited and homogenous resource for high throughput screening. Nonetheless, the pharmacological data obtained with immortalized cells do not always reflect the compounds’ desired clinical efficacy and safety^27^. The physiologically of primary cells is a much more relevant cell model than immortalized cell lines. Therefore, there has been an effort in recent years to optimize the conditions of primary neuronal cell culture, which will enable new screening assays to be developed based on primary neuronal cultures^28,29^.

Here we have designed and validated FORTIS (FluOrescence Receptor TraffIcking Screening), an efficient approach to label, monitor, and analyze synaptic efficacy and plasticity in live neurons. Genetically encoded AMPAR reporters are now available to selectively label surface AMPARs with a high signal-to-background ratio in live cells, enabling non-invasive, longitudinal monitoring of synaptic efficacy and function. As such, we used viral vectors to express recombinant AMPARs tagged with super ecliptic pHluorin (SEP, green fluorescence), and we engineered a new AMPAR subunit labeled with pHuji, pH-sensitive red fluorescence^30^. We show that FORTIS can be used in short-term and long-term experiments, allowing a temporal characterization of agents that affect AMPAR distribution. FORTIS is a versatile, fast, and cost-effective tool, and we propose that it can be used as an initial step for rapid phenotyping and drug discovery.

## Materials and methods

All the experiments were approved by the committees for ethical care and use of animals in experiments at Ben-Gurion University of the Negev (b14764_30) and the University of the Basque Country (M20/2016/001; M20/2018/296; M20/2016/019).

### Construct generation

The pHuji DNA sequence was amplified by PCR from the Addgene plasmid 61556. The pHuji sequence was introduced in-frame, downstream of the GluA1 signal peptide (22 aa), using the Gibson Assembly® Master Mix—Assembly (New England Biolabs, #E2611), and it was subcloned into the pSinRep5 plasmid under the control of the Sindbis subgeneric promoter.

### Extracellular solutions

The solutions tested to determine the most suitable solution for long-term monitoring while maintaining the pH stable were (in μM): (A) 129 NaCl, 4 KCl, 4 CaCl_2_, 10 HEPES, 10 Glucose [pH 7.4]; (B) 129 NaCl, 4 KCl, 4 CaCl_2_, 25 HEPES, 10 Glucose [pH 7.4]; (C) 129 NaCl, 4 KCl, 4 CaCl_2_, 18 NaHCO_3_, 10 HEPES, 10 Glucose [pH 7.4]; (D) 129 NaCl, 4 KCl, 4 CaCl_2_, 18 NaHCO_3_, 25 HEPES, 10 Glucose [pH 7.4].

### Fluorescence monitoring with a microplate reader

Ninety-six-well plates containing neurons maintained for 14–22 DIV were transferred to a Spark Multimode Microplate reader (Tecan). This system offers complete environmental control for live cells, with temperature, CO_2_, and humidity control, thereby guaranteeing neuronal survival for at least 50 h. After obtaining a baseline recording (5–60 min), the cultures were subjected to the experimental treatments. Fluorescence readings were obtained with a 475/535 nm (Ex−Em) excitation/emission filter for GFPs or a 485/20-535/25 filter for red fluorescent proteins. The data were post-processed using Magellan software (Tecan).

### Induction of chemical LTP and LTD

For the FORTIS experiments, dissociated primary neurons (20–24 DIV) were grown in glass-bottomed, black, 96-well plates (Cellvis, P96-1.5H-N). After replacing the culture medium with the extracellular solution (in μM: 129 NaCl, 4 KCl, 4 CaCl_2_, 18 NaHCO_3_, 10 HEPES, 10 glucose [pH 7.4]), the experiments were performed at 24 or 37 °C, with the [CO_2_] set at 5%. Baseline fluorescence intensity measurements were first obtained, after which cLTP was induced based on a protocol described previously^31^, injecting glycine to each well (final concentration of 30–500 μM) unless otherwise indicated. Briefly, neurons were treated for 5 min at 37 °C and in 5% CO_2_ with an extracellular solution containing NMDA (20 or 50 μM) along with 10 µM glycine, followed by incubation in an extracellular solution containing 4 mM MgCl_2_. The controls were treated with an extracellular solution alone.

### Statistical analysis

We did not carry out a sample size calculation or randomization of the samples. We performed some of the analysis blindly (e.g., spine density, image analysis). Cultures with a deteriorated general health were excluded from the study. All statistical analyses were performed using GraphPad Prism (version 8.00, GraphPad Software, La Jolla, CA, USA). Datasets were scrutinized for normal distribution using the Kolmogorov–Smirnov normality test in order to choose the appropriate parametric or non-parametric analysis. Data are presented as mean ± standard error of the mean (SEM) of the *n* (number of cells, cultures) indicated in each legend. All the experiments were repeated at least three times, and the presented data are the combination of the results of all the repetitions. Statistical tests (including the *p* values) used for each comparison are detailed in the figures and the figure legends.

The rest of the methods can be found in the Supplementary Material.

## Results

### Optimization of the culture conditions for 96-well plate screening

**Fig. 1:**
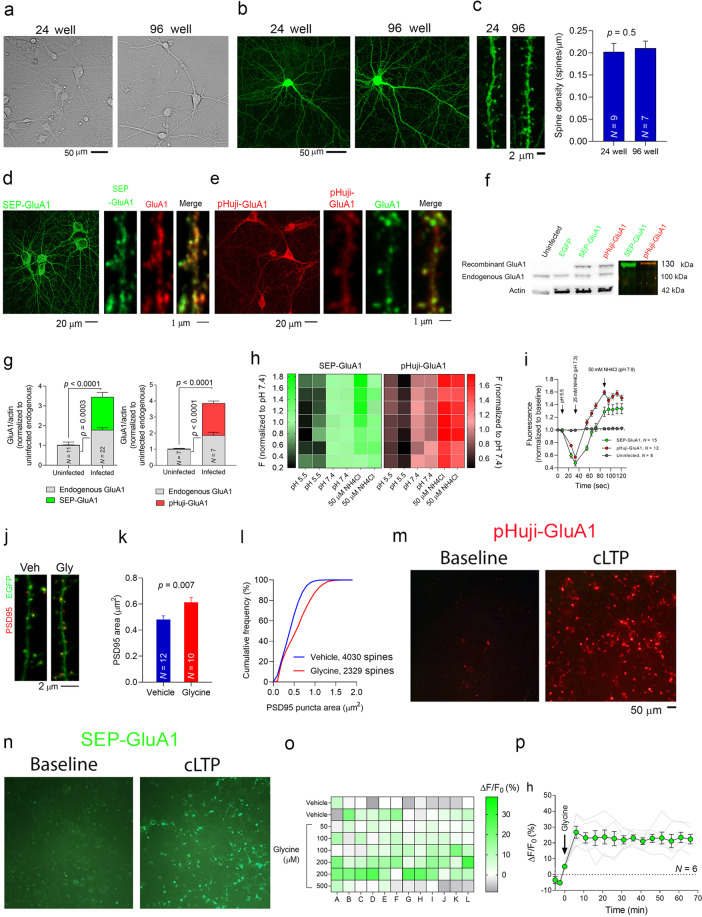
Optimization of culture conditions and characteristics of SEP- and pHuji-GluA1 AMPAR subunits. **a** Brightfield images (×20) of hippocampal neurons (20 DIV) cultured in 24- or 96-well plates. **b** Confocal projection images (×10) of hippocampal neurons expressing EGFP that were used for spine counting. **c** Left, Representative examples of confocal projection images (×63) of hippocampal neuron dendrites expressing EGFP growing in 24- or 96-well plates. Right, Quantification of the dendritic spines on hippocampal neurons growing in 24- or 96-well plates performed with Imaris software: *N* represents the number of cells, and the *p* value was determined according to the two-sided Mann–Whitney test. **d–e** Left, a confocal projection image (×40) of fixed hippocampal neurons in a 96-well plate expressing SEP-GluA1 or pHuji-GluA1. Right, a high magnification (×60, digital zoom 3) projection image of dendrites expressing SEP-GluA1 or pHuji-GluA1 immunostained with an anti-GluA1 antibody (for SEP-GluA1 in red using an Alexa 594-conjugated secondary antibody, for pHuji-GluA1 in green, using an Alexa 488-conjugated secondary antibody), to show the co-localization of the recombinant and endogenous GluA1 proteins. **f** Western blot showing the endogenous and recombinant GluA1 (SEP-GluA1 and pHuji-GluA1). In color, the lysate of infected neurons was loaded onto a polyacrylamide gel and viewed with a gel documentation system. A band of the corresponding color can be seen at the expected size. **g** Quantification of the endogenous and the recombinant GluA1 proteins. *N* represents the number of cultures, and the *p* values were determined with a Mann–Whitney test. **h** Heat maps showing the changes in SEP/pHuji-GluA1 as a function of pH and after the addition of ammonium chloride (NH_4_Cl). Each square represents the relative fluorescence in a single well (culture). **i** Real-time measurements of SEP/pHuji-GluA1 starting at pH 7.4 (time 0), after which the pH was reduced to 5.5 and then increased back to 7.3 and 7.9 with increasing concentrations of NH_4_Cl. **j** Representative projections of dendrites from hippocampal neurons expressing EGFP and treated with glycine (100 μM) to induce cLTP. The neurons were fixed for 20 min after induction, and the cultures were immunostained with the PSD95 antibody (using an Alexa 594-conjugated secondary antibody). **k** Bar graph showing the average PSD95 puncta area, where *N* represents the number of neurons, and the *p* value was determined according to the two-sided Student’s *t*-test. **l** Cumulative frequency curves showing a shift to the right in the PSD95 puncta size in glycine-treated neurons, with the number of PSD95 puncta. **m**, **n** Fluorescent images of hippocampal cultures infected with pHuji-GluA1 or SEP-GluA1, both at baseline and immediately after treatment with glycine (100 μM). **o** A heat map of the changes in SEP-GluA1 fluorescence (Δ*F*/*F*_0_, %) where each square represents a single hippocampal culture in a 96-well plate. After a 5-min baseline, cLTP was induced by injecting glycine (as indicated by the arrow). The fluorescence was measured at 485/40–528/20 (Ex−Em) every 5 min for 1 h. The color map represents the change in fluorescence 60 min after cLTP induction. **p** Changes in SEP-GluA1 fluorescence following cLTP induction. Each gray line represents the Δ*F*/*F*_0_ in a single culture (well), while the black line with the green symbol represents the average of Δ*F*/*F*_0_ (normalized to the average baseline of each culture) in the cultures treated with glycine (100 μM). The data are presented as the mean ± SEM.

Primary neurons form physiological synaptic networks, cultures, providing an experimentally accessible system to study synaptic function^32^. Thus, primary neurons remain the gold standard source material for in vitro neurobiological research. We observed that the general appearance of neurons growing in 96-well plates was similar to that of neurons growing in 24-well plates (Fig. 1a, b). The aim was to have enough neurons to ensure their survival and limit cell death^33^ while obtaining a large population of synapses^34^ to facilitate synaptic plasticity^35^. As such, we plated 15,000–60,000 cells/well onto black-walled, thin-bottomed 96-well plates in 100 μl of medium/well, and we checked their viability under a brightfield microscope. Plating 40,000 or 60,000 cells per well yielded very confluent cultures with a large proportion of viable neurons, while seeding 80,000 cells per well resulted in neuronal death (data not shown). Under these conditions (40,000–60,000 cells per well), neurons in 96-well plates were viable for 3–4 weeks in culture, allowing synaptogenesis and synaptic maturation to occur. Accordingly, the dendritic spine density in cultures grown in a 96-well plate under these conditions was similar to that of cultures growing in 24-well plates (Fig. 1c). The physiological health of the neurons was also normal, as evaluated with calcium imaging (see Suppl. Results).

We then set out to determine the optimal conditions for detecting changes in fluorescence with the microplate reader. To overcome the susceptibility of primary neurons to transfection toxicity and their inherent low transfection efficiency^36^, we used DH(26S), a neurotropic strain of Sindbis virus and a member of the alphaviruses that preferentially infects neurons over glia^37^. In our cultures, neurons represent approximately half of the cells, as witnessed by quantifying the NeuN and DAPI labeling of cells (Suppl. Fig. 1a). We first wondered if a microplate reader could detect changes in the proportion of infected neurons. Indeed, we observed a linear correlation between the proportion of infected neurons and the fluorescence detected (*F*_EGFP_: Suppl. Fig. 1b). Based on fluorescence-activated cell sorting (FACS) of infected cultures, we calculated that our purified virus titer was 1.66 × 10^8^ transducing units per ml (TU/ml). By direct visualization of fluorescent images, we confirmed that the diluted virus (3.3 × 10^6^ TU/ml) infected a large proportion of the neurons (Suppl. Fig. 1c)^37^ and that the virus is neurotoxic at concentrations above 4.0 × 10^6^ TU/ml (data not shown)^38^. Through FACS, Sindbis virus (3.3 × 10^6^ TU/ml) was seen to infect approximately 50% of the cells in the culture regardless of the number of cells seeded (Suppl. Fig. 1d), a number that corresponded to the actual proportion of the neurons in the culture (Suppl. Fig. 1a). In addition, irrespective of the virus’s titer (3.3 × 10^5^–3.3 × 10^6^ TU/ml), the fluorescence distribution of EGFP^+^ (enhanced green fluorescent protein) cells was similar (Suppl. Fig. 1e). Hence, practically all neurons in a given culture appear to be infected and the virus achieves maximal infection at a titer of 3.3 × 10^6^ TU/ml.

### Changes in surface GluA1 fluorescence can be detected with a microplate reader

To monitor the real-time fluorescence of live neurons, we first determined the optimal culture medium for scanning. Neurobasal medium alone exhibits a high level of autofluorescence in the green channel (Ex−Em 485/20–535/25 nm), regardless of the presence of serum (fetal bovine serum; FBS), cells, or phenol-red (Suppl. Fig. 1f), impeding the monitoring of GFPs. By contrast, a standard extracellular solution has an autofluorescence similar to water (Suppl. Fig. 1f). Thus, the extracellular solution can be used to monitor fluorescence when using GFPs while keeping neurons alive over a few hours. Ideally, it should be possible to monitor surface AMPARs for periods of hours or even days. As phenol red-free medium displays fluorescence in the red channel (Ex−Em 560/10–610/20) identical to an extracellular solution or water (Suppl. Fig. 1g), it might be suitable for long-term measurements. Indeed, when tdTomato using Sindbis virus was expressed in neurons maintained in phenol red-free medium, increased expression of tdTomato could be detected with a microplate reader 6 h after infection^37^ (Suppl. Fig. 1h), confirming the feasibility for long-term monitoring using red fluorescent proteins.

Fluorescent proteins with pH-sensitive fluorophores are quenched in endosomes and are valuable tools to detect protein exocytosis and endocytosis in real time^39,40^. We infected neurons with the GluA1 subunit of AMPAR fused to SEP at its N-terminal. For long-term surface GluA1 monitoring, we replaced SEP with pHuji, a red fluorescent protein with a pH sensitivity that approaches SEP^41^, creating the new fusion protein, pHuji-GluA1. We used Sindbis viral vectors to express both SEP-GluA1 and pHuji-GluA1 in neurons (Fig. 1d, e), enabling the selective labeling of surface receptors in culture^30,42^. Due to the pH-dependent visibility of both recombinant proteins, live neurons expressing SEP-GluA1 generally show weak fluorescence under basal conditions. Yet, in fixed infected neurons, SEP/pHuji-GluA1 can be seen in the soma (excluding the nucleus), dendrites, and spines (Fig. 1d, e). This fluorescence co-localized with the immunostaining of GluA1 antibodies and a band representing SEP/pHuji-GluA1 protein can be seen in western blots, in which SEP-GluA1 and pHuji-GluA1 protein bands were evident when probed with a GluA1 antibody, in addition to the endogenous protein (Fig. 1f). We also found that endogenous GluA1 expression increased by approximately 50% in infected cultures and that in infected cultures, the amount of SEP/pHuji-GluA1 expressed was equivalent to the amount of endogenous GluA1 (Fig. 1g). Together the total amount of GluA1 (endogenous plus recombinant) increased more than threefold in infected neurons (Fig. 1g), reflecting the GluA1 overexpression in our system.

As FORTIS depends on the fluorescence emitted by pH-dependent proteins, we tested different buffers for their ability to maintain a given pH for extended periods (up to 16 h) under controlled CO_2_ (5%) and temperature (24 or 37 °C) conditions in the plate reader. As expected, the addition of NaHCO_3_ to the extracellular solution was critical to maintaining the solution at a pH of 7.4. By contrast, solutions lacking NaHCO_3_ decreased their pH to acidic values (Suppl. Fig. 2a). The signal-to-background ratio (*F*_infected_/*F*_uninfected_) was lower for pHuji-GluA1 (~1.40) than SEP-GluA1 (~1.80: Suppl. Fig. 2b). However, the coefficient of variation at an extracellular pH 7.4 was lower for pHuji-GluA1 (~7%) than for SEP-GluA1 (~15%), implying a lower variability in fluorescence between cultures expressing pHuji-GluA1 (see frequency plots in Suppl. Fig. 2c). As expected, SEP/pHuji-GluA1 fluorescence was quenched in an acidic buffer (pH 5.5)^43,44^, whereas it was enhanced following treatment with ammonium chloride (NH_4_Cl, 25–50 mM: Fig. 1h–i). Thus, a microplate reader can reliably detect changes in the SEP/pHuji-GluA1 fluorescence provoked by changes in the extracellular solution. Indeed, the dynamic range of SEP-GluA1 under our experimental conditions was 3.42 (±0.50), while that of pHuji-GluA1 was 2.68 (±0.12: Fig. 1i). Thus, these AMPAR reporters generate a strong signal with a high signal-to-background ratio.

#### FORTIS can detect increases in GluA1 fluorescence attributed to chemical LTP induction

A substantial proportion of AMPARs are located within the cytoplasm of neurons, including dendrites and dendritic spines^45,46^. We reasoned that since most of the SEP-GluA1 signal is on dendritic spines^30^ and given that LTP-related increases in SEP-GluA1 can be detected in individual spines^30,47,48^, we should be able to see chemical LTP (cLTP)-like increases in SEP/pHuji-GluA1 fluorescence at the population level with a microplate reader. To ensure that our protocol indeed induces cLTP, we conducted a series of experiments employing various approaches. We induced cLTP in primary cultures with glycine (100 µM) while neurons were incubated in an Mg^2+^-free extracellular solution to prevent Mg^2+^ from blocking the activity of NMDARs^49,50^. As expected, we observed a significant increase in active (Thr286‐phosphorylated) CaMKII^51,52^ and the phosphorylation of its substrate GluA1 at Ser831, a CaMKII/PKC site^53,54^ (Suppl. Fig. 3c). These phosphorylation events were blocked by exposure to the NMDAR inhibitor APV (50 μM: Suppl. Fig. 3e). The same protocol significantly increased the labeling of active CaMKII^51,52^ in dendritic spines (Suppl. Fig. 3d) and enlarged spine heads^20,30,55^ (Fig. 1j–l). Fluorescence microscopy also demonstrated an apparent rise in SEP/pHuji-GluA1 fluorescence after cLTP induction (Fig. 1m, n). Hence, these experiments validated our cLTP protocol and indicated that this manipulation could enhance the SEP/pHuji-GluA1 fluorescence detected by FORTIS.

**Fig. 2:**
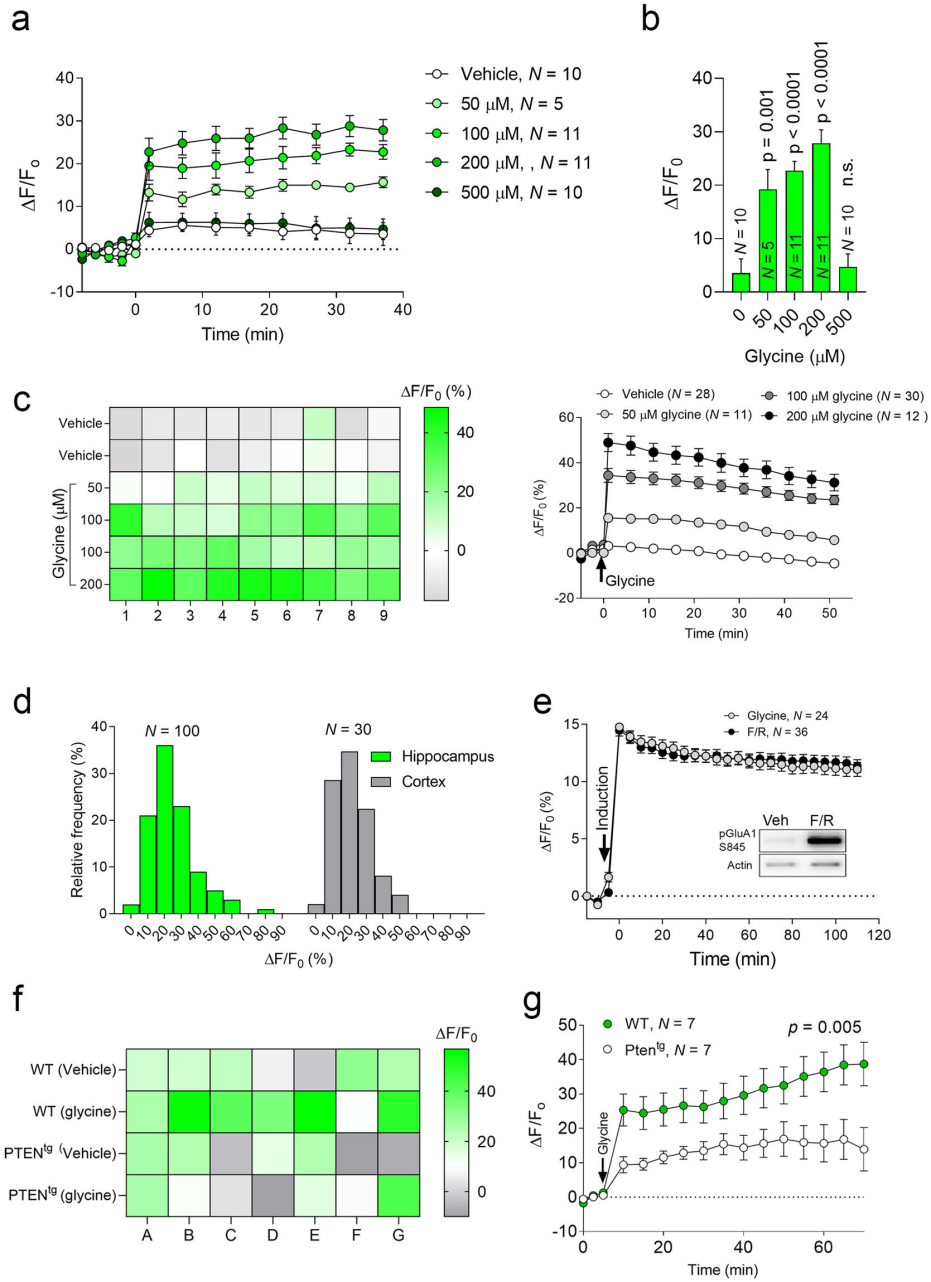
Changes in SEP-GluA1 fluorescence in response to cLTP induced under different conditions. **a** Changes in SEP-GluA1 fluorescence when different glycine concentrations were used for cLTP induction. **b** Histogram showing the Δ*F*/*F*_0_ (%) 35–40 min after cLTP induction with different glycine concentrations. **c** Left, a heat map of the changes in SEP-GluA1 fluorescence (Δ*F*/*F*_0_, %) where each square represents a single cortical culture in a 96-well plate. Right, Changes in SEP-GluA1 fluorescence when different glycine concentrations were used for induction. After the 5-min baseline, glycine was injected (indicated by the arrow), and the fluorescence was measured at 485/40–528/20 (Ex−Em) every 5 min for up to 50 min. **d** Frequency distribution graphs of Δ*F*/*F*_0_ (%) in hippocampal and cortical neurons following cLTP induction. *N* represents the number of cultures. **e** Changes in SEP-GluA1 fluorescence after cLTP were induced with either glycine (100 μM) or Forskolin (50 μM)/Rolipram (0.1 μM). **f** Heat map of the changes in SEP-GluA1 fluorescence (Δ*F*/*F*_0_, %) following cLTP induction (100 μM glycine) where each square represents a single hippocampal culture prepared from either WT or Pten^tg^ mice. **g** Changes in SEP-GluA1 fluorescence after cLTP was induced with glycine (100 μM) in cultures from WT or Pten^tg^ mice. *N* represents the number of cultures, and the *p* value was determined with two-way ANOVA, presenting the data as the mean ± SEM.

We then employed a dynamic assay to record surface AMPAR fluorescence in each well at multiple time points. By normalizing readings after drug addition to the basal readings (Δ*F*/*F*_0_), the resulting ratios reflect the increase (%) in the surface incorporation of AMPARs during the assay period. We monitored SEP-GluA1 fluorescence following cLTP induction as LTP is accompanied by the rapid insertion of AMPARs and enhanced clustering of AMPARs at the surface of dendritic membranes^56^. We replaced the medium of SEP/pHuji-GluA1-expressing neurons (24–48 h after infection, DIV 20–24) with an extracellular solution. After a baseline fluorescence recording, we automatically added glycine at different concentrations using the microplate reader’s injector. The addition of glycine produced an immediate dose-dependent increase in SEP-GluA1 fluorescence (Figs. 1o, p and 2a, b) blocked by APV (Suppl. Fig. 3e), consistent with data on LTP induced in slices and cultured cells^57–60^. Hence, it appears that FORTIS not only detects population changes in SEP-GluA1 fluorescence following cLTP induction but can also detect subtle changes induced by different doses of glycine. We observed a similar increase in pHuji-GluA1 fluorescence (Suppl. Fig. 3f). In some experiments, instead of monitoring recombinant GluA1 in live neurons, we immunostained the endogenous surface and total GluA1 in fixed cultures (Suppl. Fig. 4a). We observed with a microplate reader increases in the surface/total GluA1 fluorescence ratio following exposure to glycine (Suppl. Fig. 4b).

When we induced cLTP in cortical neuronal cultures, we observed a similar dose–response to glycine (Fig. 2c). The frequency distribution of the magnitude of Δ*F*/*F*_0_ was also indistinguishable between hippocampal and cortical neurons, showing that most cultures exhibited a 10–30% increase in fluorescence following cLTP induction (100 μM glycine: Fig. 2d). We then used a previously described chemical stimulation protocol of forskolin plus rolipram (F/R: 50 μM/0.1 μM) to provoke cLTP^61–63^. As with the glycine protocol, F/R addition induced a significant increase in SEP-GluA1 fluorescence (Fig. 2e), paralleled by a corresponding rise in Ser845 (PKA site) GluA1 phosphorylation^61,64^ (Fig. 2e). To assess if FORTIS can be used to evaluate plasticity in transgenic mice, we used hippocampal cultures from mice overexpressing phosphatase and tensin homolog (PTEN; Pten^tg^ mice) that exhibit deficits in LTP^65^. We observed a significantly smaller cLTP-related increase in SEP-GluA1 fluorescence in cultures derived from Pten^tg^ mice (Fig. 2f, g). Hence, FORTIS may be used as part of the phenotyping of transgenic mice.

#### FORTIS can detect decreases in surface AMPARs attributed to chemical LTD induction

**Fig. 3:**
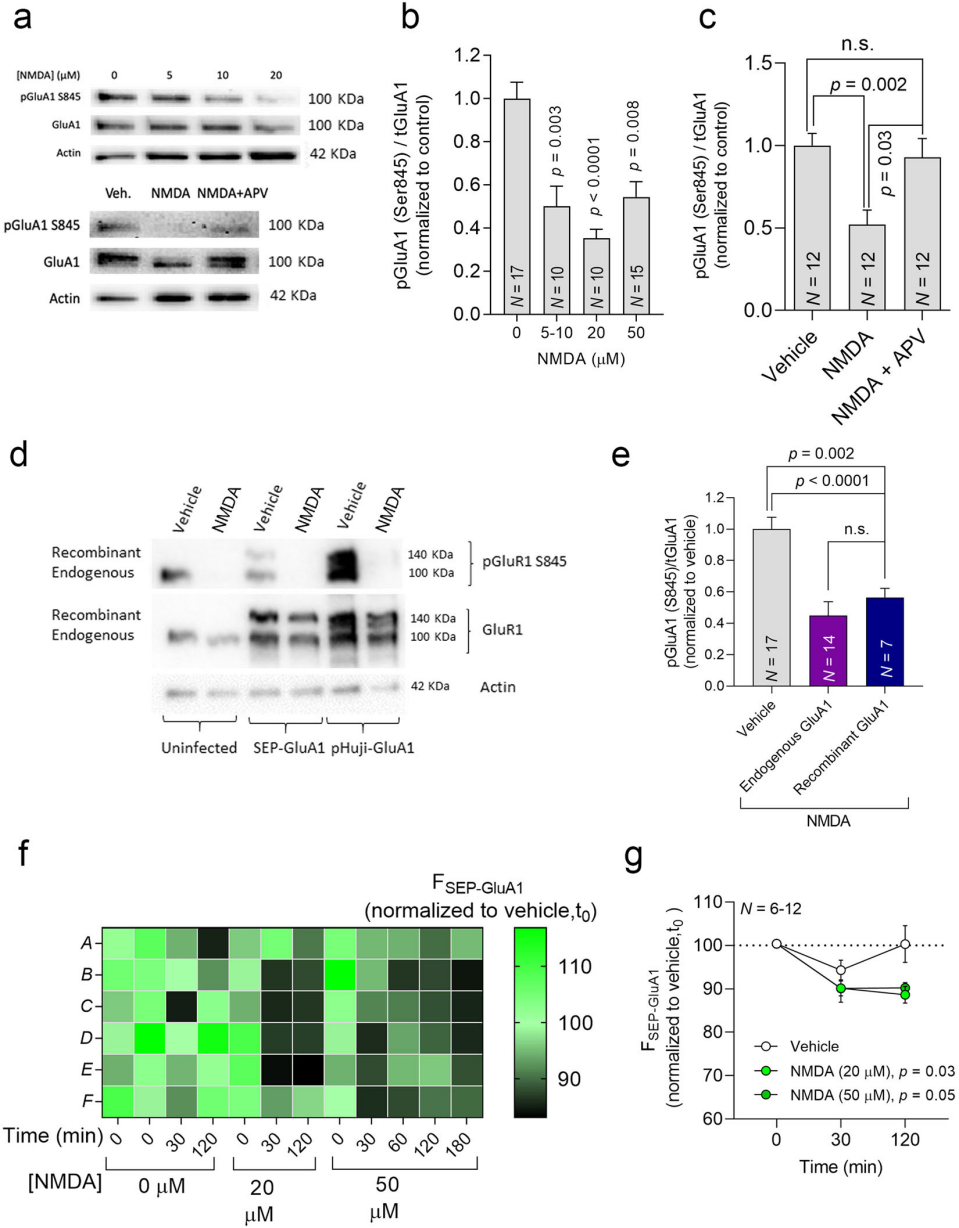
FORTIS can detect decreases in surface GluA1 following cLTD induction. **a** An immunoblot showing the levels of Ser 845 phosphorylated GluA1 or total GluA1 20 min after a 5-min exposure to different NMDA concentrations. Lower panel, in some cultures cLTD (20 μM NMDA) was induced in the presence of the NMDA inhibitor APV (50 μM). **b**, **c** Quantification of Ser 845 phospho-GluA1 following NMDA treatment with or without the inhibitor APV (50 μM). *N* represents the number of cultures, and the *p* value was determined with one-way ANOVA followed by Dunn’s multiple comparison tests. **d**, **e** Changes in endogenous and recombinant (SEP-GluA1 or pHuji-GluA1) Ser 845 GluA1 phosphorylation (normalized to vehicle) following treatment with NMDA (50 μM). *N* represents the number of cultures, and the *p* values were determined by one-way ANOVA followed by Holm–Sidak’s multiple comparisons test. **f** Heat map of the changes in SEP-GluA1 fluorescence (Δ*F*/*F*_0_, %) where each square represents a single cortical culture in a 96-well plate 120 min after a 5-min exposure to two concentrations of NMDA, as indicated. **g** Changes in SEP-GluA1 fluorescence when two NMDA concentrations were used to induce cLTD. Fluorescence was measured 30 and 120 min after 5-min treatment with NMDA. *N* represents the number of cultures, and the *p* values were determined by Dunnett’s multiple comparison test, representing the data as the mean ± SEM.

To establish the cLTD protocol for FORTIS, we first confirmed that NMDAR stimulation is accompanied by GluA1 dephosphorylation at Ser845^66^ (Fig. 3a–c). As expected, this decrease in phosphorylation was blocked by APV (50 µM, Fig. 3a, c)^61^. Crucially, the recombinant SEP-GluA1 and pHuji-GluA1 showed similar dephosphorylation to the endogenous proteins (Fig. 3d, e). After establishing the cLTD protocol, we induced cLTD in cortical neurons by applying NMDA (20 or 50 μM) for 5 min^31,67^. We detected a significant decrease in SEP-GluA1 fluorescence following this treatment, which was stable for at least 2 h after applying NMDA (Fig. 3f, g). We obtained similar results from hippocampal neurons (Suppl. Fig. 4c), and thus FORTIS can be used with a microplate reader to detect reductions in surface AMPARs triggered by cLTD stimulation.

#### FORTIS can identify known cognitive enhancers

**Fig. 4:**
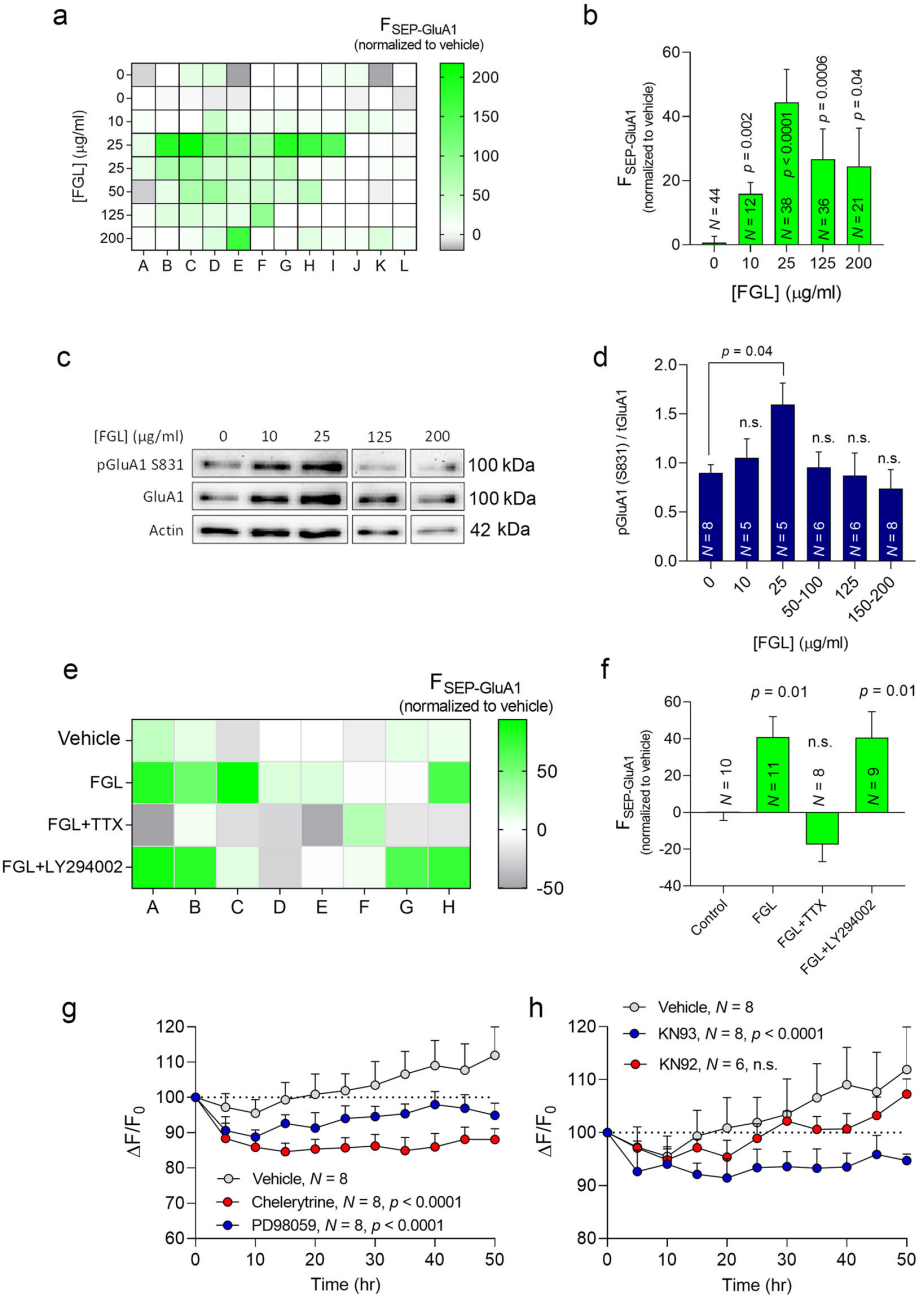
FORTIS can detect the activity of the cognitive enhancer FGL. **a** Heat map of SEP-GluA1 fluorescence where each square represents a single hippocampal culture in a 96-well plate, measured following a 48 h exposure to different doses of FGL (10, 25, 50, 125, and 200 µg/ml). **b** Fluorescence intensity as a function of FGL dose. **c** Representative immunoblots show the levels of S831 phosphorylated GluA1 in dissociated neurons treated with different concentrations of FGL for 48 h. Actin was used as a loading control. **d** Quantification of pGluA1/tGluA1 following treatment with different concentrations of FGL. **e** Heat map of SEP-GluA1 fluorescence measured following a 48 h exposure to FGL (25 µg/ml) in combination with TTX (3 μM) or the PI3K inhibitor LY294002 (10 μM). **f** Fluorescence intensity as a function of FGL, TTX, and LY294002 treatment. *N* in **b**, **d**, and **f** represents the number of cultures. Statistical significance in **b**, **d**, and **f** was calculated according to the Mann–Whitney test followed by Tukey’s multiple comparisons post hoc tests, and the data are presented as the mean ± SEM. **g**, **h** Long-term monitoring of pHuji-GluA1 fluorescence following the administration of PD98059 (25 μM), chelerythrine (10 μM), KN93 (20 μM), or KN92 (20 μM). The *p* values were determined with two-way ANOVA.

We tested our system with two drugs known to increase the amount of AMPAR at synapses and enhance cognitive function in rodents. The synthetic mimetic peptide of the neural cell adhesion molecule (NCAM), FGLoop (FGL), was explicitly engineered to mimic the functional interaction between NCAM and the fibroblast growth factor receptor (FGFR)^18,68,69^. FGL initiates a signaling cascade that translates into persistent CaMKII activity and facilitates the synaptic delivery of AMPARs during synaptic plasticity^16,18,70^, and enhances learning and memory in various experimental paradigms, supporting the strong link between AMPAR activity and cognitive enhancement^18,71,72^. We treated SEP-GluA1-expressing neurons with FGL (10–200 μg/ml) and observed a dose–response increase in SEP-GluA1 fluorescence 24 h later, with the peak at 25 μg/ml (Fig. 4a, b). We also found a dose-dependent increase in phospho-GluA1 with the highest phosphorylation observed at 25 μg/ml (Fig. 4c, d), consistent with observations in organotypic cultures^18^. When the neurons were exposed to both FGL and TTX (3 µM), the increase in SEP-GluA1 fluorescence in the presence of FGL was abrogated (Fig. 4e, f). Hence FGL appeared to require a neuronal activity for the delivery of AMPARs to synapses. By contrast, the PI3K inhibitor had no effect on FGL activity, further evidence that FGL does not modulate AMPAR delivery via the PI3K pathway^18^ (Fig. 4e, f). Together, these findings suggest that FORTIS can detect the effects of a drug known to facilitate AMPAR surface delivery.

Conversely, we inhibited three signaling pathways known to facilitate AMPAR trafficking in neurons expressing pHuji-GluA1: the MAPK, PKC, and CaMKII pathways^18,73–75^. Exposing the cells to PD98059 (25 µM), a potent inhibitor of MAPK kinase (MEK)^75^, or chelerythrine (5 µM), a general PKC inhibitor^73,76^, reduced pHuji-GluA1 fluorescence over 50 h (Fig. 4g). Similarly, the inhibition of CaMKII with KN93 (20 µM) ^18,77^ but not exposure to its inactive derivative KN92 (20 μM) induced a significant long-term decrease in pHuji-GluA1 (Fig. 4h). These data suggest that FORTIS is sensitive and sufficiently specific to detect changes in surface AMPARs arising from well-established drugs with known effects on AMPAR transmission.

**Fig. 5:**
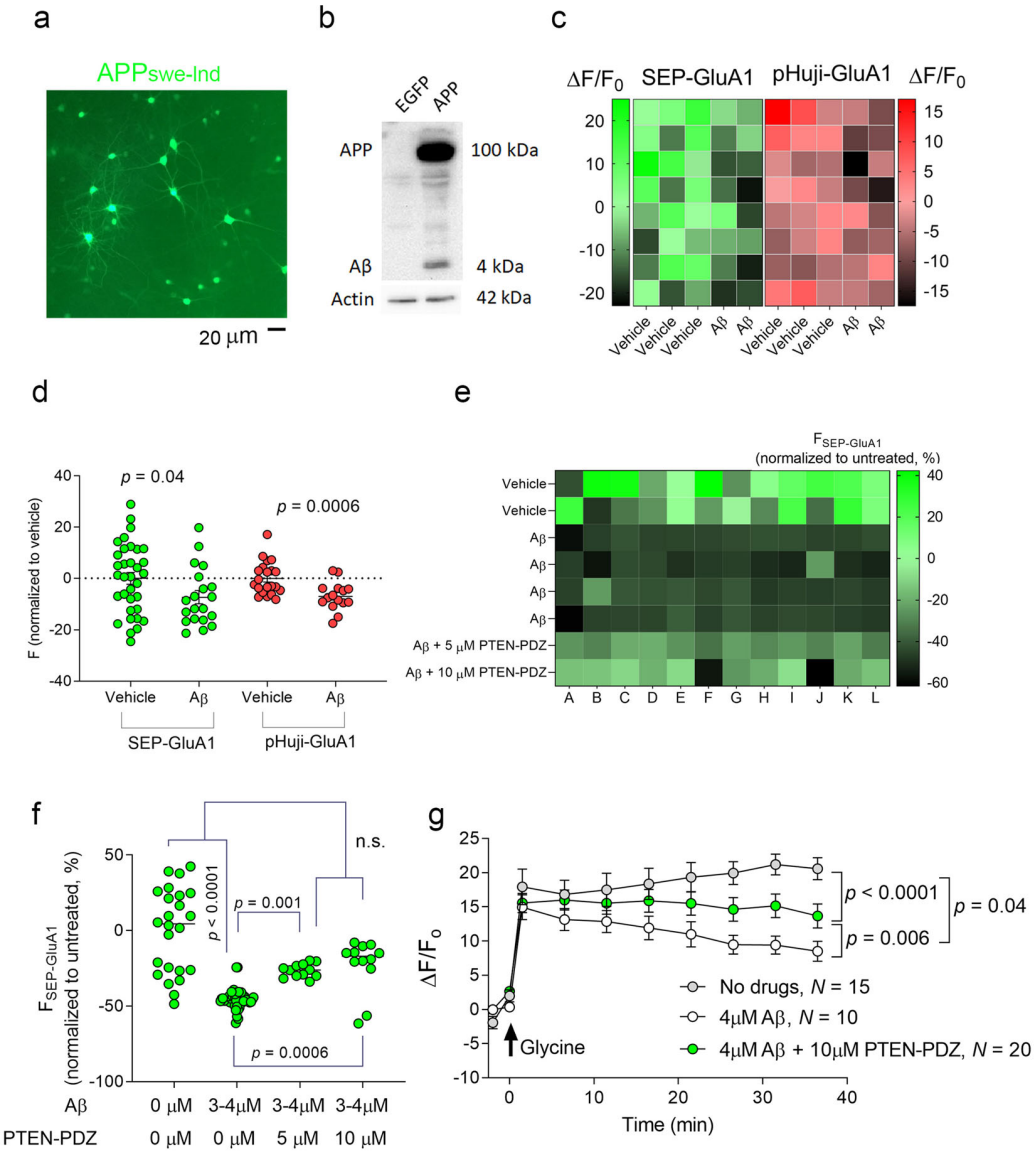
FORTIS can detect endocytosis induced by Aβ. **a** Hippocampal neurons overproducing Aβ42 due to the expression of APP_swe/lnd_. The medium of these cultures that contains Aβ42 was used to induce AMPAR endocytosis in **c** and **d**. **b** A western blot of lysates of neurons expressing either EGFP or APP_swe/lnd_ and probed with an anti-β-Amyloid antibody only recognizes an APP and Aβ band in the cultures expressing APP_swe/lnd_. **c** A Heat map of SEP-GluA1 and pHuji-GluA1 fluorescence where each square represents a single cortical culture in a 96-well plate following treatment with medium taken from neurons expressing APP_swe/lnd_ and secreting Aβ42 (see **a**). **d** Quantification of the experiment shown in **c**, showing the individual relative fluorescence (each circle represents a single culture) following treatment with the Aβ42-containing medium. Statistical significance was calculated according to the Mann–Whitney test. **e** Heat map of SEP-GluA1 relative fluorescence where each square represents a single hippocampal culture in a 96-well plate following treatment with synthetic Aβ42 (3 or 4 μM), with or without preincubation with the PTEN-PDZ peptide (5 or 10 μM). **f** Quantification of the experiment shown in **e**, showing the individual relative fluorescence (each circle represents a single culture) following treatment with synthetic Aβ42 and the PTEN-PDZ peptide. Statistical significance was calculated according to the Mann–Whitney test. **g** cLTP was induced in cultures treated with Aβ42 (4 μM), with or without preincubation with the PTEN-PDZ peptide (10 μM). Statistical significance was calculated according to two-way ANOVA followed by Tukey’s multiple comparisons post hoc tests, and the data are presented as the mean ± SEM.

Based on the current hypothesis regarding the molecular basis of memory failure in Alzheimer’s disease (AD), soluble assemblies of the amyloid-β peptide (Aβ) are responsible for synaptic malfunctions that provoke a range of deficits from mild cognitive impairment to dementia^17,78–82^. Indeed, Amyloid β (Aβ) induces decreases in AMPAR efficacy indicative of synaptic depression as an early AD event^17,80,82,83^. We first confirmed that FORTIS could detect Aβ-induced AMPAR endocytosis, for which we used Aβ secreted by neurons following the virally driven expression of a mutant form of Amyloid precursor protein (App, human App with the Swedish/London double mutation, App_swe/lnd_-IRES-EGFP, Fig. 5a, b)^17^. We replaced half of the culture medium of neurons expressing either SEP-GluA1 or pHuji-GluA1 with medium from neurons expressing App_swe/lnd_ (final Aβ concentration: 10.29 ± 5.5.01 pg/ml). As a control, we used a medium from neurons expressing EGFP following infection with the same type of virus. Following a 24 h Aβ treatment, we observed a significant decrease in both SEP-GluA1 and pHuji-GluA1 fluorescence (Fig. 5c, d), suggesting that both recombinant GluA1s react similarly to Aβ application. If FORTIS can detect Aβ-induced reductions in surface AMPAR, it may also detect drugs that prevent this effect. Thus, SEP-GluA1-expressing neurons were exposed to a peptide (“PTEN-PDZ”; 5 or 10 µM) that blocks the interaction of PTEN with PDZ proteins, thereby preventing AMPAR endocytosis and synaptic depression, rescuing cognitive impairment in Alzheimer’s model mice^17,70^. We incubated neurons with the peptide for 1 h at 37 °C and, after obtaining baseline measurements, we injected freshly prepared synthetic Aβ protofibrils (3–4 µM)^17^ and measured the fluorescence. The synthetic Aβ elicited a decrease in SEP-GluA1 fluorescence (Fig. 5e, f). Yet, SEP-GluA1 fluorescence was rescued in the cultures treated with both Aβ and the PTEN-PDZ peptide, confirming previous electrophysiological findings^17^. As Aβ assemblies alter synaptic plasticity by inhibiting LTP in hippocampal neurons in vitro^17,79^, we induced cLTP with glycine (100 μM). In neurons treated with Aβ, we noticed a weaker cLTP-like increase in SEP-GluA1 fluorescence, which was partially rescued by the “PTEN-PDZ” peptide (Fig. 5g). These findings suggest that FORTIS can detect drugs that impede the endocytosis of AMPAR.

## Discussion

This work describes the development and validation of FORTIS, an assay to assess surface AMPARs in live populations of primary neurons. FORTIS detects subtle changes in surface AMPARs, including synaptic plasticity-like changes. In addition, FORTIS is successful in detecting the effects of drugs on surface AMPARs. AMPARs are a target of interest for drug discovery and development as they play a critical role in synaptic plasticity mechanisms that may underlie learning and memory^16,18,84,85^. By employing FORTIS and rapidly testing modulators of AMPAR trafficking, we were able to test their effect while avoiding the standard time-consuming experiments required to achieve cellular or synaptic resolution. This study represents the first attempt to use SEP-GluA1 for the rapid, non-invasive neuronal populations’ characterization with a microplate reader. The addition of the new red pH-sensitive pHuji-GluA1 reporter, combined with instruments that integrate sensitive detectors and incubation under controlled CO_2_, temperature, and humidity conditions, allows us to monitor surface AMPARs over temporal scales of seconds to days. As described here for AMPARs, SEP or pHuji can be fused to subunits of other receptors that control synaptic function, such as NMDA receptors, which will enable their live monitoring in different populations of neurons. These features make FORTIS compatible with studies of long-term responses to pharmacological treatments at a high temporal resolution, permitting cause-and-effect relationships to be determined. The length of the measurements may be extended by using less cytotoxic viral vectors or endogenous GluA1 tagged with pH-sensitive reporters^86^. Since FORTIS uses a microplate reader without imaging, its spatial resolution is limited, and it cannot distinguish extrasynaptic from synaptic receptors. Nevertheless, a significant advantage of FORTIS is that it monitors thousands of neurons simultaneously, and, therefore, it is substantially faster than the methods used traditionally to detect changes in AMPARs. For example, patch-clamp recording is carried out on one neuron at a time, while imaging can be performed on a few dendrites and spines, considerably limiting the throughput of these methods. Moreover, FORTIS can test dozens of drugs simultaneously, which is impossible with traditional approaches. In addition, and unlike the aforementioned approaches, FORTIS does not require expertise other than that required for culture preparation, and it does not require special equipment, such as electrophysiology set-ups or expensive microscopes.

Together, FORTIS may provide useful measurements for the functional screening of new synaptic modulators. FORTIS can be used to classify neuronal responses to multiple systematic perturbations, and it can be used in basic science, translational research, and drug development. For example, applying FORTIS to cell models of AD may help find novel compounds or targets that restore aberrant synaptic function, which could serve as the basis for new mechanism-based treatments. The development of positive modulators of AMPARs holds excellent promise to discover safe, effective treatments for memory and cognitive impairments. Together, the combined features of FORTIS may enhance the accessibility of drug discovery in the neuroscience community, which could stimulate drug discovery for neuropsychiatric disorders.

## Supplementary information

SUPPLEMENTAL MATERIAL

Suppl Fig 1

Suppl Fig 2

Suppl Fig 3

Suppl Fig 4
