# Supplementary material for: FORTIS: a live-cell assay to monitor AMPA receptors using pH-sensitive fluorescence tags
Source: Transl Psychiatry. 2021 May 27;11:324. doi: 10.1038/s41398-021-01457-w (PMC8160262; doi:10.1038/s41398-021-01457-w)
Supplement: Supplementary file 1 — SUPPLEMENTAL MATERIAL [file 41398_2021_1457_MOESM1_ESM.docx]

Supplementary information:

Supplementary materials and Methods

***Neuronal Cultures.*** Primary hippocampal neurons were prepared from mice or Wistar rats as described elsewhere ^1, 2^. In brief, neurons were dissociated from the E15 mouse or E18 Wistar rat hippocampus or cortex and plated in 96-well optical plates. Neurons were maintained at 37 °C in a 5% CO_2_ incubator, and 20% of the media was changed every 7 days. A similar protocol was followed for small-scale cultures in 12-24 well plates on poly-L-lysine coated coverslips. Cultures were treated with cytosine β-D-arabinofuranoside (final concentration 5 μM: Sigma-Aldrich, C1768) to diminish glial proliferation (7 days in vitro -DIV). To prevent an edge effect due to thermal gradients and differential evaporation rates ^3^, peripheral wells, we loaded with water. For the experiment involving Pten^tg^ mice, Pten^tg^ and WT embryos were distinguished by weight, and their genotype was later confirmed by PCR using the primers: Forward-T7: 5′-CCGCTAATACGACTCACTATAGGG-3′ and Reverse-T7: 5′-TCATCTCGGCTCCATCGTTT-3′ ^2, 4^.

***Virus preparation and infection of neurons.*** The virus was prepared as described previously ^2, 5^. Briefly, 5-10 μg of the plasmid of interest (pSinRep5) and helper plasmid (pDHtRNA) were linearized using NotI and XhoI restriction enzymes (Thermo Scientific, FD0595 and FD0694), respectively. In vitro RNA transcription of the linearized plasmids was performed using the mMESSAGE mMACHINE® SP6 Transcription Kit (Life Technologies, AM1340), and the RNA was recovered by the phenol-chloroform method, except that precipitation was performed by adding 1 volume of isopropanol. RNA was centrifuged for 15 minutes at 14,000 rpm (4 °C), the pellet was resuspended in 10-15 ml nuclease-free water and stored at -20 °C for transfection. For each nucleofection, a total of 10 ×10^6^ cells were pelleted by centrifugation and resuspended in 100 µl Nucleofector solution, along with 10 μg of the transcript of interest and 10 μg of the helper transcript. The RNA-containing cell suspension was transferred to a cuvette (Lonza) and nucleofected in the Amaxa Nucleofector, following a specific protocol for the BHK-21 cell line. Immediately afterward, the cells were transferred and plated in a 150 mm dish and maintained at 37 °C in 5 % CO_2_. The efficiency of transfection was checked 24h later by examining the fluorescence and cytopathic effects in the cells, such as elongation and detachment from the substrate ^6^. Finally, 48-72h post-nucleofection, the culture medium of dying cells was collected and viral particles were concentrated on a 4 ml 20% sucrose cushion by ultracentrifugation with an SW28.1 rotor at 25,000 rpm. The supernatant was discarded, and the pellet was resuspended with 200 μl of neurobasal medium with 5% FBS. The resuspended virus was aliquoted into 5-20 μl aliquots and stored immediately at -80 °C.

***Western Blotting.*** Cell plates were washed once with ice-cold PBS before the cells were lysed by scrapping in lysis buffer (10 mM HEPES pH 7.4, 150 mM NaCl, 10 mM EDTA, 1 % Triton X-100, protease inhibitor cocktail tablets "Complete mini EDTA-free" and phosphatase inhibitor cocktail tablets "PhosSTOP": Roche, 04693159001 and 04906837001). The lysates were collected and centrifuged at 13,000 rpm for 5 minutes at 4 °C and the supernatants were used for protein quantification using the BCA protein assay (Pierce, 23227). Standards of bovine serum albumin (BSA) prepared at known concentrations were used to determine the concentration of the protein extracts. Equal protein quantities were prepared in 4x sample loading buffer, and samples were boiled to 95 °C for 5 minutes immediately before electrophoresis. Proteins (10 µg per lane) were separated according to their molecular weight by SDS-PAGE in a Mini PROTEAN Tetra Cell Vertical Electrophoresis system (Bio-Rad) and in 1x running buffer (25 mM Tris [pH 8.3], 192 mM glycine, 0.1% SDS in dH_2_O). Following electrophoresis, the proteins were transferred from the gel to a PVDF membrane (pore size 0.45 µm: Amersham Hybond, 10600023) in the Mini Trans-Blot Cell transfer system (Bio-Rad) and in 1x transfer buffer (25 mM Tris [pH 7.6], 192 mM glycine, 20% methanol in dH_2_O) for 90 minutes at 400 mA. The membrane was then stained with Ponceau S (Sigma Aldrich, P3504) to confirm the successful transfer of the proteins, and non-specific binding was blocked with 5 % (w/v) non-fat dry milk powder in TBS-T (20 mM Tris, 137 mM NaCl, 0.1% Tween-20 in dH_2_O [pH 7.6]) for 1 hour at room temperature with gentle shaking. To detect phosphorylated proteins, 5 % (w/v) Phospho BLOCKER™ Blocking Reagent in TBS-T was used (Cell Biolabs, AKR-103). The membranes were probed with a specific primary antibody diluted in blocking solution overnight at 4 °C with gentle shaking, washed three times for 5 minutes with TBS-T, and subsequently incubated with the corresponding horseradish peroxidase (HRP)-labeled secondary antibody in blocking solution for 1 hour at room temperature. The membranes were again washed as described above, and the antibodies bound to their target proteins were detected by enhanced chemiluminescence (Luminata Forte Western HRP Substrate, Millipore, MIWBLUF0100) in a MyECL Imager (Thermo Scientific) and quantified by densitometry using the Quantity One software (Bio-Rad).

***Calcium imaging in 96-well plates.*** As a measure of spontaneous neuronal activity, calcium-imaging experiments were performed using the FDSS/µCELL (Functional Drug Screening System: Hamamatsu Photonics), and with Oregon GreenTM 488 BAPTA-1 AM (Thermo Fisher Scientific, 06807) as a calcium indicator. To this end, 96 well-plated neurons (DIV 20-24) maintained in Neurobasal medium supplemented with B27 were loaded for 30 minutes with the calcium indicator (final concentration 50 ng/µl) at 37 °C, in an atmosphere of 5% CO_2_. Pluronic F-127 (dissolved in 10 % DMSO: Sigma Aldrich, P2443) was used at a final concentration of 0.4 % along with Oregon GreenTM 488 BAPTA-1 AM (Thermo Fisher Scientific, # O6807) to facilitate dye loading. The cells were then washed, and for fluorescent measurements at 480-540 (Ex-Em) at 37 °C and in an atmosphere of 5% CO_2_, they were placed in the extracellular solution (in mM): 129 NaCl, 4 KCl, 4 CaCl_2_, 25 NaHCO_3_, 1 NaH_2_PO_4_, 10 Glucose [pH 7.4]). Spontaneous activity was recorded at a frequency of 0.5-2 Hz.

***Morphology.*** To assess the overall morphology of 96-well plated dissociated neurons, cells (DIV 20-24) were infected with Sindbis virus (EGFP) for 24 hours, and their dendrites and dendritic spines were then visualized. The cells were fixed in fresh 4% paraformaldehyde (PFA) in PBS for 10 minutes at room temperature and washed three times with PBS. Cells were covered with Prolong Gold Antifade Reagent (Thermo Fisher Scientific, P36934), and after 24 h, they were visualized on a Zeiss LSM880 Airyscan confocal microscope equipped with an Argon 488 nm laser line. A tile-scan application was used to obtain images of whole neurons (10x) or dendrites (63x). Spine quantification was performed using Imaris 7.2 software (Bitplane Inc.), and the spine density was calculated for each dendrite by dividing the number of spines by the corresponding dendritic length.

***Immunohistochemistry.*** The protocol followed for cLTP induction was described previously with minor changes ^7^. Specifically, cultured neurons were incubated for 20 minutes at 37 °C and 5% CO_2_ in an extracellular solution (in mM: 129 NaCl, 4 KCl, 4 CaCl_2_, 10 HEPES, 10 Glucose [pH 7.4]) containing glycine (200 μM). Controls were incubated in glycine-free extracellular solution (vehicle). For immunofluorescence experiments, hippocampal neurons seeded on 12 mm diameter coverslips (Menzel-Gläser, CB00120RA1) were fixed in 4% PFA in PBS for 10 minutes at room temperature and washed three times with PBS. The cells were then incubated in blocking solution (3% BSA, 5 % goat serum, 0.1 % Triton X-100 in PBS; except when performing anti-NeuN and anti-GFAP staining when they were blocked with 5% BSA, 4% goat serum, 0.1 % Triton X-100 in PBS) for 1 hour at room temperature. Fixed cells were incubated with specific primary antibodies diluted in blocking solution overnight at 4 °C, washed three times for 5 minutes with PBS, and subsequently incubated with the corresponding fluorochrome-conjugated secondary antibodies in blocking buffer for 1 hour at room temperature. Finally, the cells were mounted using Prolong Gold Antifade Reagent (Thermo Fisher Scientific, P36934), and images were acquired on a ZEISS LSM 900/880 with Airyscan 2 using ZEN (ZEISS Efficient Navigation software). The acquisition of SEP/pHuji expressing neurons was performed using two different objectives: LD Plan-NEOFLUAR 20x/0.4 Corr and LD LCI Plan-APOCHROMAT 63x/1.2 with immersion oil (ImmersolTM 518F, Zeiss). Single field images of 1024 x 1024 pixels were collected using 488 nm laser to visualize SEP and 522 nm laser to visualize pHuji. Multiple stage positions were collected using a motorized stage with a step-size of 5-10 μm.

To assess the PSD95 puncta size or the phosphorylated CaMKII in dendritic spines after cLTP induction, hippocampal neurons were infected with the Sindbis virus for 24 hours to express EGFP, allowing dendritic spines to be visualized. Fixed cells were incubated for 1 hour in blocking solution and probed overnight at 4 °C with a specific antibody against phosphorylated CaMKII (T286) or PSD95. After washing the cells with PBS, they were probed for 1 hour with a fluorochrome-conjugated secondary antibody in a blocking solution. Finally, the coverslips were mounted, and confocal images were obtained using a 63x oil-immersion objective. The Spots mode was used to detect spine heads, and a mask was applied to the green or red channel with Imaris software to yield the area of PSD-95 puncta and the sum intensity of pCaMKII.

***Flow Cytometry.*** Neurons were harvested by adding Trypsin-EDTA at 0.05%, the medium was removed by aspiration, and the cells were washed twice with PBS. Subsequently, 100 μl/well of Trypsin-EDTA (0.05%) was added, and the cells incubated for 5 minutes at 37 ⁰C, stopping the reaction by adding 200 μl of DMEM + 10% FBS. The neurons were collected in 1.5 ml tubes and centrifuged at 500 g for 5 minutes. The supernatant was decanted and the pellet was resuspended in 200 μl of PBS + 5% FBS. To determine the presence of fluorescent proteins in the neurons (infected neurons), 95,000 cells per condition were analyzed by Cytoflex S (Beckman Coulter). A negative control (uninfected neurons) was used for the cell population of interest and to determine the background levels of autofluorescence.

***Amyloid β.*** Water-soluble Aβ42 assemblies were prepared as described previously ^2^. Briefly, lyophilized Aβ42 peptide (1 mg: Keck Facility, Yale University) was dissolved in 50 μl DMSO to obtain Aβ42 aggregates, to which 800 μl of H_2_O and 10 μl of 2 M Tris-base solution (pH 7.6) was added. The solution was then incubated at 24-26 °C for 5 min and used immediately. To quantify the Aβ content in the culture medium of APP_swe/lnd_-EGFP-Sindbis infected neurons by ELISA, the culture medium from cortical rat neurons (20 DIV) infected with APP_swe/lnd_-EGFP sindbis virus for 24 h was collected and stored at -20 °C. The Aβ was quantified in triplicate in samples from three independent experiments using the Human Aβ-42 ELISA kit (Invitrogen, Cat. # KHB3441) according to the manufacturer's instructions. The standard curve ranging from 0 to 1000 pg/ml was prepared fresh.

***Virus titration***. The infection of primary neuronal cultures was performed by adding a medium containing an aliquot of a purified virus to the cell plate under different working conditions ^8^. Primary neurons were seeded at 100,000 cells per well in a 96-well plate, and neurons (14-20 DIV) were infected with the corresponding virus at different dilutions (1:50, 1:100, 1:500, and 1:1000). Negative controls were not infected with the virus. The cells were then harvested and analyzed by flow cytometry to detect the fluorescent protein associated with the expression of the virus (positive cells: infected neurons that express our protein of interest). The quantification of the virus at the different dilutions was determined as:


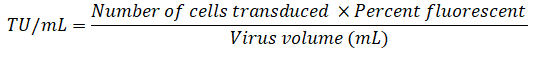


***Antibodies.*** The primary antibodies used were raised against*:* Glial Fibrillary Acidic Protein (GFAP: Dako, Z0334), NeuN (polyclonal: Synaptic Systems, 266 006), phosphoGluA1 S831 (Millipore #AB5847), phosphoGluA1 S845 (Invitrogen #OPA1-04118), GluA1 (Cell signaling, 13185S), GluA1 (Cell signaling 13185S, and Abcam #ab31232), phospho-CaMKIIa, T286 (Millipore #05-533), CaMKIIa (Sigma Aldrich #C6974), PSD95 (NeuroMab, 75-028), β-actin (Cell signaling,4970S) and β-Amyloid (6E10, BioLegend #803001). The secondary antibodies used were*:* anti-mouse and anti-rabbit IgG HRP-linked secondary antibodies (Cell signaling, 7076S, and 7074S), goat anti-Chicken Alexa Fluor® 633, (Invitrogen, 10444562), goat anti-Mouse IgG (H+L) Cross-Adsorbed Alexa Fluor 594 and 488 antibodies (Thermo Fisher Scientific, A-11005 and A-21121), Goat anti-Rabbit IgG (H+L) Cross-Adsorbed Alexa Fluor 488 and 594 Secondary Antibody (Thermo Fisher Scientific, A-11008 and A-11012).

***Drugs.*** The drugs used in this study were: Biccuculine methiodide (Sigma-Aldrich #B7561), Chelerythrine (Sigma-Aldrich #C2932), DL-AP5 (Tocris Bioscience #0105), Forskolin (Sigma-Aldrich #F3917), Glycine (Bio Lab Ltd_UN warehouse #071323), KN-92 (Millipore Sigma #422709), KN-93 (Sigma-Aldrich #K1385), LY-294002 (Sigma-Aldrich #L9908), NMDA (Sigma-Aldrich #M3262), PD-98259 (Sigma-Aldrich #P215), Picrotoxin (Tocris Bioscience #1128), Strychnine (Sigma-Aldrich # S0532-5G), Tetrodoxin (TTx: Tocris Bioscience #1078), BAPTA-AM (Sigma Aldrich A1076), Rolipram (Sigma Aldrich, R6520), LY294002 (Sigma Aldrich, 440202).

**Supplementary Results**

We assessed the health of neurons in 96-well plates through their response to depolarization. We first treated the cultures (14 DIV) for 30 minutes with the calcium indicator Oregon Green^TM^ 488 BAPTA 1-AM (Suppl. Fig. 3a), after which we replaced the Neurobasal medium with the extracellular solution. We recorded spontaneous calcium activity on a plate reader. We observed the expected synchronized bursting network activity in regular neuronal cultures ^9^ that could be blocked by the sodium channel blocker, Tetrodotoxin (TTX, 3 µM: Suppl. Fig. 3a). The brief and transient depolarization triggered by 50 mM KCl results in an increased synaptic activity of cultured neurons accompanied by the insertion of AMPARs into synapses ^10, 11^. Accordingly, when we depolarized the neurons with 50 mM KCl we observed an enhanced calcium signal (Suppl. Fig. 3a). In neurons expressing SEP-GluA1, we saw stronger fluorescence in dendrites after a similar stimulation (Suppl. Fig. 3b), following previous experiments with neurons cultured in 24 well plates ^11, 12^. These observations imply that neurons in cultures in 96-well plates are physiology equivalent to those in 24 well plates and that changes in surface GluA1 can be detected with a plate reader.

Supplementary figure legends

**Fig. S1. Fluorescence measurements with a microplate reader. a. Left,** A confocal projection image (10x, zoom 2) of a typical primary hippocampal culture staining with DAPI (blue, nuclei), with a NeuN antibody as a marker for neurons (red, Alexa 594 conjugated secondary antibody) and with GFAP antibody as a marker of astrocytes (green, Alexa 488 conjugated secondary antibody). **Right.** Quantification of the NeuN and DAPI positive cell density to evaluate the proportion of neurons in the cultures: *N* represents the number of cultures. **b.** **Left,** A heat map of the fluorescence (a.u.) as a function of the proportion of infected neurons expressing EGFP. **Right,** Graph showing a linear correlation between the proportion of infected neurons expressing EGFP and the relative fluorescence in the plate reader. **c.** A low magnification (10x) image of EGFP expressing neurons in a 96-well plate used to monitor EGFP fluorescence. **d.** FACS analysis of the proportion of EGFP+ cells as a function of the number of cells seeded: N represents the number of cultures. **e.** FACS analysis of the number of EGFP+ cells at different virus concentrations. The images were obtained with the FlowJo software, and the data are presented as the mean ± SEM. **f.** **Left,** Autofluorescence of the culture medium (NB, neurobasal medium; FBS, fetal bovine serum) and the extracellular solutions (ECS) in the green channel in the absence of cells. Each dot represents the fluorescence in a single well (culture). **Right,** Fluorescence emitted by the culture medium or ECS plus the fluorescence emitted from cells expressing SEP-GluA1. Each dot represents the fluorescence in a single well. **g.** Autofluorescence in the red channel in the absence of cells. Each dot represents the fluorescence in a single well. **h. Left,** hippocampal neurons expressing dtTomato were used to test the feasibility of long-term monitoring of red fluorescence. **Right,** The change in fluorescence following viral infection to express dtTomato.

**Fig. S2. Measurement of pH and fluorescence. a.** Comparison of the pH at different extracellular solutions (ECS) during 16 hours. **b. Left,** Heat maps showing the relative SEP-GluA1 and pHuji-GluA1 fluorescence in infected and uninfected neurons in ECS, where each square represents the relative fluorescence in a single well. **Right,** Quantification of the fluorescence of infected and uninfected neuronal cultures. The data are shown as the mean ± SEM, where N is the number of cultures. **c.** Frequency distribution of relative SEP-GluA1 and pHuji-GluA1 fluorescence, where N represents the number of cultures.

**Fig. S3. Chemical LTP. a. Left,** Hippocampal neurons cultured (21 DIV) in a 96-well plate loaded with the Oregon Green BAPTA-1 calcium indicator. **Right,** Fluorescence was measured every six milliseconds (RFU, relative fluorescence units) on a microplate reader. The traces reflect the calcium signal of a single hippocampal culture in a 96-well plate showing the spontaneous activity (top), the activity abolished by TTX (3 µM, middle), and the enhanced activity following KCl (50 mM, bottom) treatment. **b.** SEP-GluA1 expressing hippocampal neurons (21 DIV) before and after treatment with KCl. The data are presented as the mean ± SEM. **c. Left,** Immunoblot probed with antibodies against phosphorylated GluA1 (S831) and CaMKII (T286). Both blots were stripped and re-probed with antibodies recognizing the total GluA1 and CaMKII. **Right,** Quantification of the changes in phosphorylation of GluA1 and CaMKII, 20 min after cLTP induction (100 μM glycine) in the presence or absence of the NMDAR inhibitor APV (50 μM). *N* represents the number of cultures, and the p-values were determined with a Mann-Whitney test. **d.** **Left,** Confocal projection images (x63) of dendrites from neurons expressing EGFP, treated with glycine to induce cLTP and immunostained with an anti-phospho-CaMKII antibody that was detected with an Alexa-594 conjugated secondary antibody. **Middle,** Bar graphs of the phospho-CaMKII fluorescence intensity quantified in dendritic spines. N is the number of spines, and the p-value was determined with a Student t-test. **Right,** Cumulative frequency of phospho-CaMKII fluorescence intensity, showing a shift to the right in the spines of cultures treated with glycine relative to the control cultures. **e.** Changes in SEP-GluA1 after cLTP induction in neurons treated with glycine in the presence or absence of APV (50 μM). P values were determined by Two-way ANOVA followed by Tukey's multiple comparisons test. **f.** Changes in pHuji-GluA1 after cLTP induction in neurons treated with glycine**.**

**Fig S4. FORTIS detects decreases in SEP-GluA1 following cLTP and cLTD induction.** **a.** Confocal images (10x) of fixed hippocampal neurons immunostained for surface GluA1 (secondary antibody, Alexa 594) and total GluA1 (Secondary antibody, Alexa 488). **b. Left,** Heat maps of the surface and total GluA1 fluorescence showing increased surface GluA1 fluorescence following cLTP induction read by the microplate reader. **Right,** Quantification of the surface GluA1/total GluA1 ratio following cLTP induction. N is the number of cultures, and the data are presented as the mean ± SEM. **c.** **Left,** a Heat map of changes in SEP-GluA1 fluorescence (ΔF/F_0_, %) where each square represents a single hippocampal culture in a 96 well plate 120 minutes after a 5-minute treatment with two concentrations of NMDA, as indicated. **Right**, Changes in SEP-GluA1 fluorescence where three NMDA concentrations were used to induce cLTD. The fluorescence was measured 120 minutes after the 5-minute treatment with NMDA. Each dot represents the relative fluorescence in a single culture, and the p-values were determined with a Dunn's multiple comparisons test.

**References**

1. Sánchez-Puelles, C.*, et al.* PTEN activity defines an axis for plasticity at cortico-amygdala synapses and influences social behavior. **30**, 505-524 (2020).

2. Knafo, S.*, et al.* PTEN recruitment controls synaptic and cognitive function in Alzheimer's models. *Nat Neurosci* **19**, 443-453 (2016).

3. Lundholt, B.K., Scudder, K.M. & Pagliaro, L.J.J.o.b.s. A simple technique for reducing edge effect in cell-based assays. **8**, 566-570 (2003).

4. Sanchez-Puelles, C.*, et al.* PTEN activity defines an axis for plasticity at cortico-amygdala synapses and influences social behavior. *Cereb Cortex* **30**, 505-524 (2020).

5. Knafo, S.*, et al.* Facilitation of AMPA receptor synaptic delivery as a molecular mechanism for cognitive enhancement. *PLoS Biol* **10**, e1001262 (2012).

6. Malinow, R.*, et al.* Introduction of green fluorescent protein (GFP) into hippocampal neurons through viral infection. *Cold Spring Harb Protoc* **2010**, pdb. prot5406 (2010).

7. Lu, W.-Y.*, et al.* Activation of Synaptic NMDA Receptors Induces Membrane Insertion of New AMPA Receptors and LTP in Cultured Hippocampal Neurons. *Neuron* **29**, 243-254 (2001).

8. Sena-Esteves, M. & Gao, G.J.C.S.H.P. Titration of lentivirus vectors. **2018**, pdb. prot095695 (2018).

9. Penn, Y., Segal, M. & Moses, E. Network synchronization in hippocampal neurons. *Proceedings of the National Academy of Sciences* **113**, 3341-3346 (2016).

10. Pickard, L.*, et al.* Transient synaptic activation of NMDA receptors leads to the insertion of native AMPA receptors at hippocampal neuronal plasma membranes. *Neuropharmacology* **41**, 700-713 (2001).

11. Appleby, V.J.*, et al.* LTP in hippocampal neurons is associated with a CaMKII-mediated increase in GluA1 surface expression. *Journal of Neurochemistry* **116**, 530-543 (2011).

12. Mueller, B.H.*, et al.* Sigma-1 receptor stimulation attenuates calcium influx through activated L-type Voltage Gated Calcium Channels in purified retinal ganglion cells. *Experimental Eye Research* **107**, 21-31 (2013).
