# Supplementary figures and images for: FORTIS: a live-cell assay to monitor AMPA receptors using pH-sensitive fluorescence tags
Source: Transl Psychiatry. 2021 May 27;11:324. doi: 10.1038/s41398-021-01457-w (PMC8160262; doi:10.1038/s41398-021-01457-w)

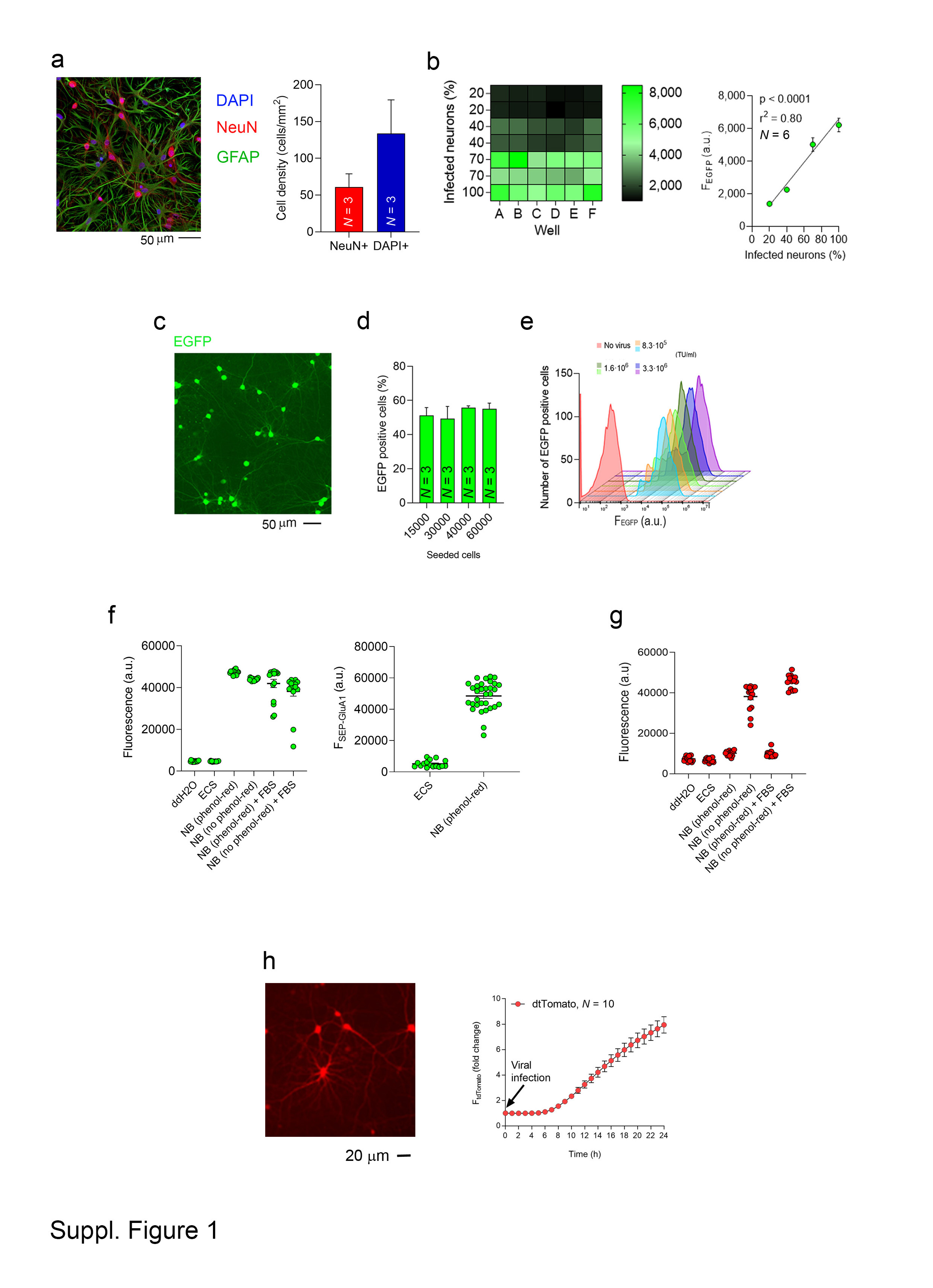

Supplement: Supplementary file 2 — Suppl Fig 1 [file 41398_2021_1457_MOESM2_ESM.jpg]

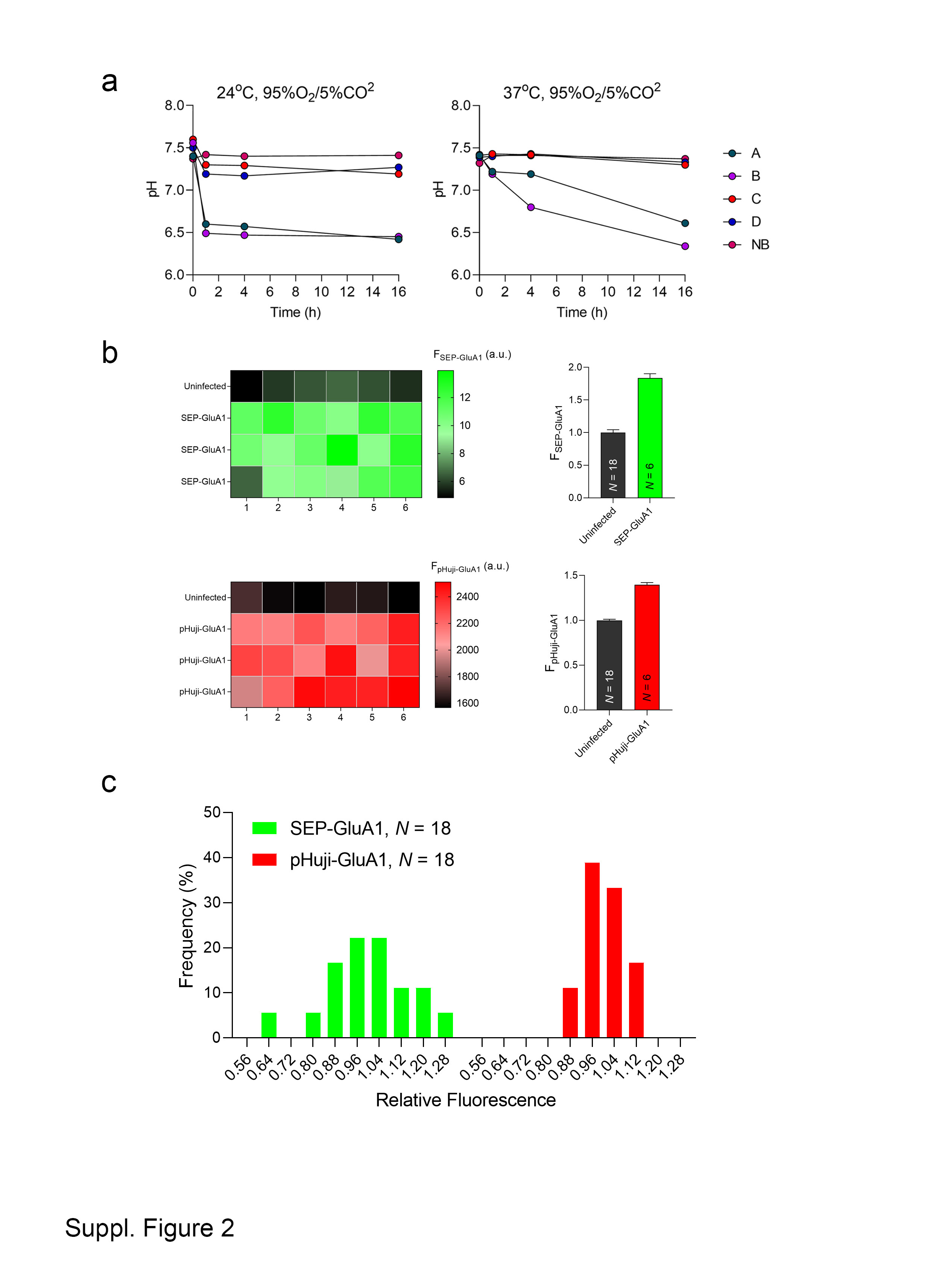

Supplement: Supplementary file 3 — Suppl Fig 2 [file 41398_2021_1457_MOESM3_ESM.jpg]

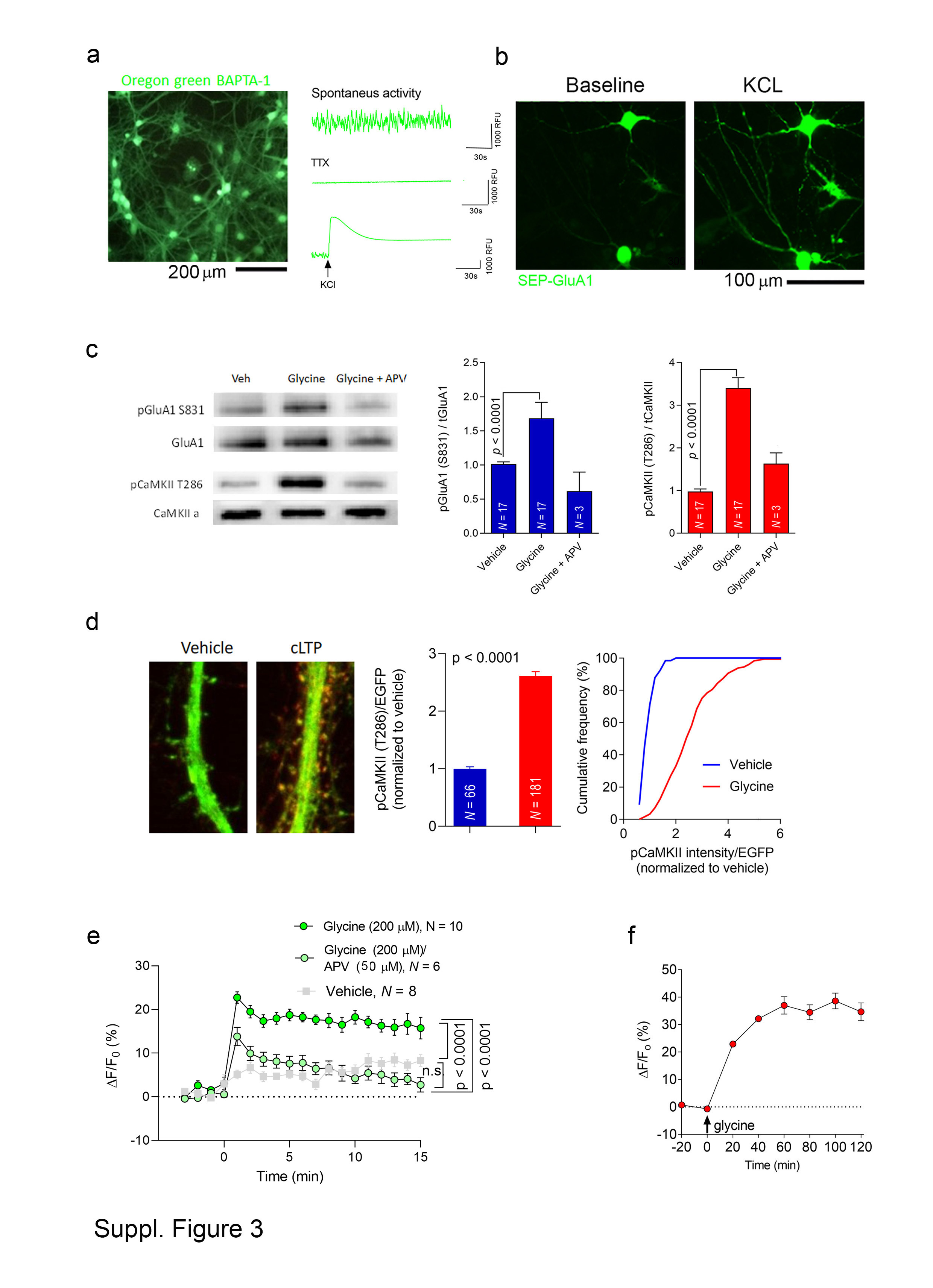

Supplement: Supplementary file 4 — Suppl Fig 3 [file 41398_2021_1457_MOESM4_ESM.jpg]

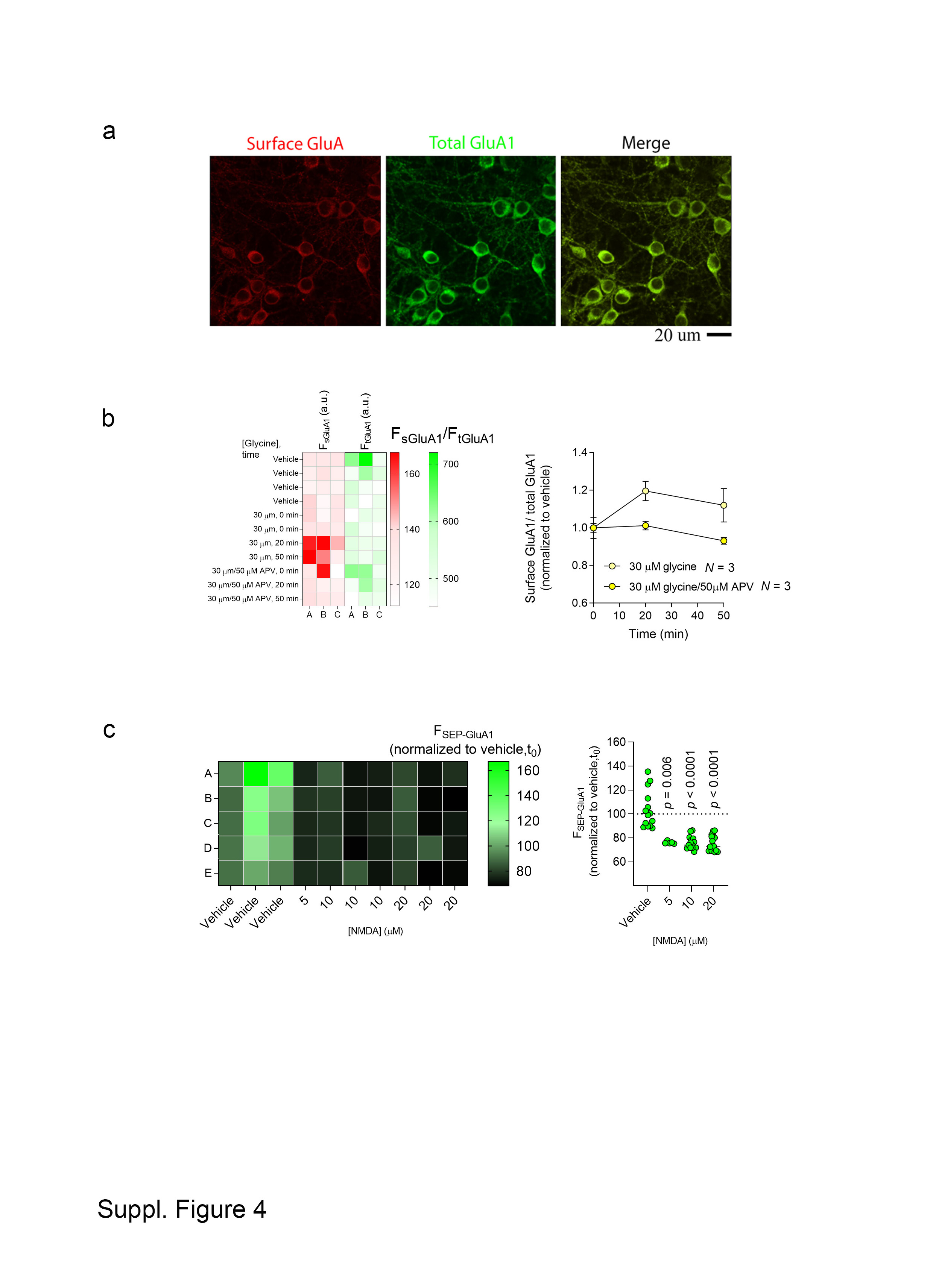

Supplement: Supplementary file 5 — Suppl Fig 4 [file 41398_2021_1457_MOESM5_ESM.jpg]
